# Buildings behaving badly: a behavioral experiment on how different motivational frames influence residential energy label adoption in the Netherlands

**DOI:** 10.1007/s10901-016-9500-y

**Published:** 2016-02-01

**Authors:** Jillian Student, Elissaios Papyrakis, Pieter van Beukering

**Affiliations:** 1 Environmental Policy and Environmental Systems Analysis Groups, Wageningen University, Droevendaalsesteeg 4, 6708 PB Wageningen, The Netherlands; 2 International Institute of Social Studies (ISS), Erasmus University Rotterdam, Kortenaerkade 12, 2518 AX The Hague, The Netherlands; 3 School of International Development, University of East Anglia, Norwich, NR4 7TJ UK; 4 Institute for Environmental Studies, Vrije Universiteit Amsterdam, De Boelelaan 1087, 1081 HV Amsterdam, The Netherlands

**Keywords:** Building energy efficiency, Energy conservation, Energy efficiency, (Residential) energy label, Motivating conservation behavior, Motivational (message) framing, Social norms, The Netherlands

## Abstract

Heating buildings contributes to approximately 36 % of Europe’s energy demand and several EU member states have adopted mandatory energy labels to improve energy efficiency by promoting home weatherization investments. This paper focuses on the perception of the energy label for residential buildings in the Netherlands and the role of different frames (egoistic, biospheric and social norms and neutral frames) in motivating adoption of energy labels for housing. We used a behavioral email experiment and an online survey to investigate these motivational factors. We find that biospheric frames are weaker than the other three motivational frames in terms of engaging interest in the energy label, but that the biospheric frame results in higher willingness to pay (WTP) for the energy label. We also find that age (rather than income) correlates with higher WTP for home energy labels.

## Introduction

While attention to both global and local environmental problems has been increasing, peoples’ actions do not always reflect these improvements in environmental awareness and per capita energy consumption is still on the rise (Steg 2008). In Europe, heating buildings is the source of 36 % of CO_2_ emissions (Agentschap NL, 2011). Heating and cooling buildings contribute to two major environmental problems, i.e. resource depletion and climate change. Energy conservation is a critical component of the EU’s plan to curtail emissions (by at least 20 % by 2020 compared to 1990 levels, see Agentschap NL, 2011; Europa 2015). Residential buildings are especially relevant for saving energy in Europe since 60 % of the estimated energy savings potential for buildings comes from private homes (IDEAL EPBD 2011).

Promoting conservation through energy efficient buildings is an obvious way of addressing this issue. However, many individuals do not know how efficient their building is and what they can do to maximize their home’s conservation potential. Energy labels are an important information/market instrument that can help promote awareness on the current level of buildings’ energy efficiency, indicate ways to improve efficiency and create explicit value for energy savings (Banerjee and Solomon 2003; Truffer et al. 2001). The residential energy label program is similar to that of energy labels for appliances (e.g. washing machines and refrigerators). The label gives a score between A(++) and G (A++ meaning a highly energy-efficient home and G a highly inefficient home). Unlike the appliance energy label, the residential label also provides information on how to improve energy efficiency and thereby the label score (e.g. conservation behaviors such as insulating a house). The goal of the program is twofold: (1) provide information on the building’s current energy efficiency and (2) suggest potential measures to improve efficiency and promote energy conservation. Residents benefit by having better insight into their homes’ energy efficiency and what they can do about improving it. Improved efficiency can enhance their comfort level and reduce their spending on energy. Also, as awareness of energy efficiency and energy costs increase, a better label can improve the property’s market value as well as provide buyers and renters a better indication of how efficient the building is (Brounen and Kok 2011a, b).

New knowledge (e.g. in the form of energy labeling) can help change attitudes, promote energy savings and influence consumers’ demand for housing by considering such information (Steg and Vlek 2009; Stern et al. 1987; Abrahamse et al. 2007). However, new knowledge does not always ensure behavioral changes in terms of energy conservation (Abrahamse et al. 2007). People tend to be reluctant to adjust their behavior when sacrifices are needed (e.g. initial monetary/inconvenience costs). Furthermore, consumers may not be able to accurately assess the short-term investment costs and discomfort against the long-term benefits (Griskevicius et al. 2010; Steg 2008).

The energy label for residential buildings is not widely used in practice although consumers could benefit from more energy efficient homes. Sixty-five percent of Dutch homes were built before 1980 and these buildings are 50 % less efficient than homes built in the last 10 years (Brounen et al. 2012). Furthermore, approximately 5 % of the Dutch household budget is spent on heating and electricity (CBS 2009). In 2009, average costs per household for gas (heating) ranged from €1200 to €2800 annually depending on the level of heating efficiency (Brounen and Kok 2011a; Brounen et al. 2012). The Netherlands pays the fourth highest amount for gas (heating) per kWh in Europe (EEP 2013). Moreover, lower income households budget a proportionally higher part of their income for energy costs (CBS 2009; Brounen and Kok 2011a). Nonetheless, the energy label has met considerable resistance and lack of interest in the Netherlands.

The opposition to the label in the Netherlands is attributed to unclear and lenient policy, negative media attention and distrust (Brounen and Kok 2011a). First, the label was officially voluntary until January 1, 2013. Lack of incentives for adopting a label and absence of label enforcement meant consumers could and still can easily avoid getting the label (Murphy and Meijer 2011). Second, unclear policy goals and unsatisfactory implementation led to critical reports from several real estate experts (e.g. the Dutch Association of Realtors, the Homeowners Association and the Renters Association) (Brounen and Kok 2011a). For example, the methodology of calculating the score was not standardized, leading to different label ratings for the same house. The consumer advocacy TV program, Radar, investigated and exposed these problems (see Radar 2007, 2008). The industry’s lack of support coupled with negative media attention contributed to lowering consumer confidence in the label; the decreasing label adoption rate (while in 2008 25 % of houses sold had a label, this fell to 15 % after the airing of Radar) demonstrates some distrust in the energy label (Brounen and Kok 2011a).

Since January 2015, some changes have been made to the residential energy label program. All houses receive a provisional label based on the year it was built, the surface area and building type. However, as with the old system, the label is mandatory only when selling or renting houses and is valid for 10 years. Failing to provide the label entails the risk of being fined up to €405 (Rijksoverheid 2015). However, people have the opportunity to get the label up until the last minute to avoid the fine, which limits potential buyers’ and renters’ access to information. To get the definite label, individuals fill in an online form, which is remotely assessed by professionals (Rijksoverheid 2015). No one comes to inspect the house, which can affect the label’s credibility as information can be intentionally or unintentionally misrepresented or inaccurate. Majcen et al. (2013a) conclude that thorough in-person inspections are needed if the label is to correctly represent residential buildings’ energy efficiency; moreover, accurate calculations are difficult to obtain when inspection is not conducted in a thorough manner. For example, even slight changes in insulation estimates (U-value) result in very different demand levels and energy label calculations. The current changes to the energy label program do not incorporate these insights.

Past research has assessed the role of the energy label as a tool for conservation. Murphy and Meijer (2011) and Murphy et al. (2012) analyzed the energy label in the Netherlands; their findings suggest that the label has failed to reach the policy measure’s target group and resulted in minimal emissions reductions. Majcen et al.’s (2013a, b) research suggests the energy label score can predict gas consumption to a degree, but that theoretical estimates of energy use in terms of the energy label are not indicative enough and actual energy consumption averages at each label level should be used instead; moreover, the finding that a good label does not necessarily mean lower energy consumption reflects the findings of similar studies (e.g. Backhaus et al. 2011). On a more positive note, Brounen and Kok (2011a, b) utilized information from real estate and energy label databases to show that homes with a good label (C or higher) have a shorter selling time (on average 24 days shorter) and a higher selling price (approximately 3 % more) than comparable houses with a red label (i.e. after taking into account differences in location, quality and year of construction). They also discovered that a better energy label score (e.g. A) does correlate with a lower average monthly gas costs for comparable houses, but that most people are neither interested nor aware of their energy consumption (with young high-income individuals having the lowest awareness levels) (Brounen et al. 2011). Thus, many households are unaware of how their household could conserve energy by having a better label score. Moreover, the Energy Center of the Netherlands (ECN) performed an EU-wide household analysis of consumer motivation and barriers related to the energy label. Their findings suggest that *how* the message for the label is framed (what motivational incentive and context are used as well as understandability) is as crucial as *what* kind of information is provided (Backhaus et al. 2011). For example, they find that people are particularly concerned about presentation and comfort when renovating their home. People are also more likely to adopt energy saving suggestions during renovations when these are presented in a comprehensible manner. They suggest exploring how to present the label as an avenue for further research.

Peoples’ interests relate to the persistent question of how to motivate households to get a label and lower their energy consumption. Griskevicius et al. (2008) find that economists and policymakers often rely on costly financial incentives or developing expensive campaigns in order to mitigate climate change. In the Netherlands, governmental information campaigns “assume that people are persuaded more by arguments based on egoistic considerations” and design their campaigns to appeal to this type of audience (De Groot and Steg 2009, p. 64). De Groot and Steg (2009, p. 64) further claim that “these campaigns often fail to promote sustainable behavior” because they do not appeal to other values and potentially cheaper and more effective methods could be used to entice pro-environmental behavior. For example, Griskevicius et al. (2008) and Cialdini and Schultz (2004) claim that social norms are an underused tool for promoting climate change abatement and decreasing energy consumption.

Our paper extends the current literature by making use of a behavioral email experiment and online survey to assess the role of different motivational frames (*egoistic/*self-interest, *biospheric*/environmental, *social norms* and *neutral*/information only) in stimulating behavioral change and, in particular, adoption of energy labels for conservation. Furthermore, this is the first study to our knowledge that explores consumers’ perceived value of an energy label through their willingness to pay (WTP) estimates and links this with their underlying socio-economic characteristics.

## Theoretical framework

As energy labels try to solve an environmental problem, the assumption is that one needs to point out environmental values to motivate individuals to get labels in order to conserve more energy. However, the situation is often more complex in reality. Improving the effectiveness of information tools is linked to understanding and tapping into the right motivational frame of consumers (Steg and Vlek 2009). For this reason, the behavioral influence of available information (e.g. in terms of encouraging energy efficiency) is likely to be mediated by the way such information is framed (see Tangari and Smith 2012). Furthermore, evidence on environmental behavior also points to the fact that, although people may claim to care about the environment in general, they are often inconsistent in terms of their actual environmental behavior; for example, Steg and Vlek (2009) found that, in the Netherlands, even if people actively recycle or eat less meat, they do not necessarily conserve more energy than the average person. Thus, environmental messages may not necessarily motivate adoption of energy labels.

This research was inspired by the social normative research led by Cialdini et al. (1990) (see also Cialdini and Schultz 2004; Cialdini et al. 2006; Goldstein et al. 2008; Griskevicius et al. 2008; Nolan et al. 2008) that employed different motivational frames to see what framing would elicit the highest response to an environmental problem. For example, in one experiment the researchers left California residents door hangers with different motivational frames (biospheric, egoistic, social norms, social responsibility and neutral (information only) framing) to encourage individuals to use fans instead of air-conditioning (Cialdini and Schultz 2004). In this experiment, as well as other ones which use motivational frames to encourage towel and linen reuse (Goldstein et al. 2008), these researchers found that social norms were an effective (and non-costly) frame to encourage pro-environmental behavior. Due to the ongoing problem of active energy label adoption, we wanted to research whether social norms or other motivational frames were more effective in eliciting a response from Dutch residents to get an energy label. In our case, an email was sent to participants about the energy label (framed in four different ways) querying their interest in receiving an energy label. Four motivating frames we explore in this study are (1) *egoistic* motivations, (2) *biospheric* (environmental) concerns, (3) *social norms* and (4) *neutral* framing. Our research uses these frames to analyze which message(s) motivate(s) interest in the energy label in an online behavioral experiment. A separate online survey is used to understand the consumers’ profile, WTP and their perception of the energy label as well as environmental problems. *Egoistic* motivations relate to self-enhancement values (that place focus primarily on self and self-oriented goals); these are explained by the theory of planned behavior that claims behavior is guided primarily by one’s attitude and personal goals balanced with perceptions on behavioral consequences and social norms, which do not necessarily need to contradict one’s personal aspirations (Ajzen 1991; Abrahamse and Steg 2009; Bamberg and Möser 2007; Steg and Vlek 2009). When *egoistic* motivations are prominent, the literature advocates making green products cheaper and more efficient, as well as offering financial incentives, such as subsidies and tax deductions (Griskevicius et al. 2010). Tangari and Smith (2012) find environmental messages to be more effective when they place emphasis on anticipated (monetary) benefits in the shorter term. This is because there is often a negative relationship between self-enhancement values and environmental action, with the latter often being framed as requiring some sort of sacrifice (Schultz 2001; Corraliza and Berenguer 2000; Schultz and Zelenzy 2003).

*Biospheric* values relate to the concern for the present and future well-being of the environment and biosphere (Schultz and Zelenzy 2003; De Groot and Steg 2010). To highlight *biospheric *values, information should make these people aware of the consequences of their actions on the physical and social environment and stress personal responsibility to act (Griskevicius et al. 2010; Steg and Vlek 2009).

*Social norms* relate to the belief of what is commonly done by others (e.g. peers, local community) and the importance of conforming with a certain expected social behavior (Griskevicius et al. 2010). The focus theory of normative conduct places emphasis on the ability of *social norms* to predict and alter human behavior (Cialdini et al. 2006). According to this theory, *social norms* relate to individual’s desire to self-identify with a social group that one’s behavior is being compared to (Griskevicius et al. 2008). When there is uncertainty (e.g. in terms of the timing and type of energy price shocks and climate change impacts), individuals tend to look at others to determine how they should act (i.e. determine what is the ‘socially appropriate’ behavior) (Cialdini et al. 2006).

Similar to the fan versus air-conditioning door hanger experiment (Cialdini and Schultz 2004; Nolan et al. 2008), a *neutral* (information only) framing is also used in our study—this is for sake of comparison against the other frames that incorporate a motivational message (*egoistic*, *biospheric* and *social norms*).

## Methods

### Participants

For the purposes of conducting our study, we approached 4000 randomly selected clients of a Dutch utility company in the spring of 2012, 3985 of which actually received the online survey. It is unknown how many individuals opened their email. A sub-sample of 3188 were invited to participate in the behavioral email experiment by email. The reason for excluding part of the sample from the behavioral experiment was to enable comparison between those who had received information through the behavioral experiment with those who did not. Sample composition is described in more detail in Sects. 3.2 and 3.3. A sub-sample of 333 individuals (i.e. response rate 10 %) responded to the email experiment that preceded the survey (see Sect. 3.2). Our sample for the online survey consists of 611 individuals who agreed to participate (see Sect. 3.3) (i.e. a response rate close to 15 %). Table 1 illustrates some key characteristics of the survey sample (of 611 respondents) compared to the general Dutch population.

**Table 1:** Comparison of survey sample versus Dutch population averages

	Sample average	Dutch average
Annual gas consumption	1904.6 m^3^	1600 m^3^ Milieu Centraal (2012) 1850 m^3^ CBS (2012a)
Annual electricity consumption	3523.24 kWh	3500 kWh CBS (2012a)
Living situation	13.4 % Renters 86.6 % Homeowners	44.2 % Renters 55.1 % Homeowners 0.7 % Unknown CBS (2012b)
Household size	2.66	2.20 CBS (2015)
Age	41–50 or 4.40 (1–6 scale)	41.10 Central Intelligence Agency (2012)

**Fig. 1:**
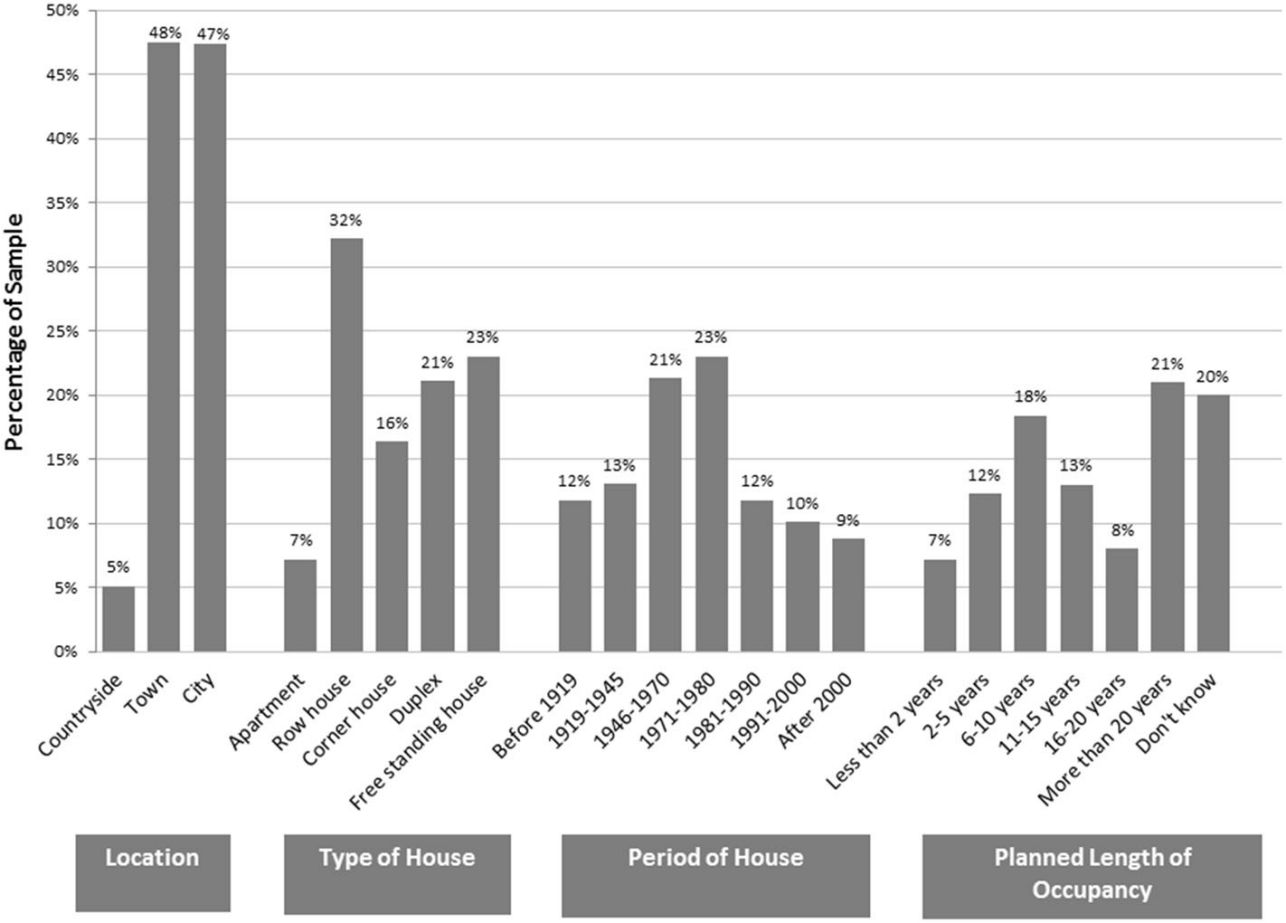
Sample living conditions (*N* = 611). *Note* Some values may not add up to 100 % due to rounding

The energy supplier’s clients are located throughout the Netherlands with diverse living situations (Fig. 1). The majority of the survey respondents are involved in making decisions about energy in their households (27.1 % were solely responsible, 71.8 % were jointly responsible). Female participants accounted for 33.5 % of respondents and male participants 66.5 %. Although the sample was randomly selected, most of the respondents were homeowners. Information about actual annual gas and electricity consumption was provided by the utility company and was contrasted against self-reported survey data.

### The behavioral email experiment

The experiment explored the role of different motivational frames (provided in an email message) in promoting interest in residential energy labels. Participants received one of the following messages that encouraged energy label adoption: (1) improving the environment (the *biospheric* frame), (2) saving money and improving comfort (the *egoistic* frame), (3) behaving similarly to other consumers (the *social norm* frame) and (4) *neutral* information about the role of an energy label in conserving energy (see Table 2 for the four email subject lines used in the experiment). The layout of the 4 emails was similar to the door hangers in Cialdini’s experiment (e.g. Cialdini and Schultz 2004). For each message, different framing was applied to motivate lowering energy consumption and to briefly describe the label’s benefits. To determine if individual were interested in the label based on the manner it was framed, a weblink was provided where they could sign-up for an online indication label (which is comparable to an unverified definite label) when it was available (for more specific information on the messages in Dutch please contact the corresponding author). A fifth group did not receive a behavioral experiment email and were only approached to complete the online survey at the same time as the other four groups. All five groups approached were of equal size (797 individuals).

**Table 2:** Motivational frames

Motivational frame	Email subject title
Egoistic	Save money by conserving energy (Bespaar geld door zuiniger om te gaan met energie)
Biospheric	Protect the environment by conserving energy (Bescherm het milieu door energie te besparen)
Social norms	Join your neighbors in conserving energy (Vergelijk uw energieverbruik met uw buren)
Neutral information	Energy conservation (Energiebesparing)

Dutch text in parentheses

To measure individuals’ responses to the different motivational frames, a follow-up link to sign-up for the online energy label was included in the email for interested recipients. If the individuals chose to follow the link, they were then asked to confirm their interest (through a ‘yes’ or ‘no’ response) and provide contact information. Only individuals interested in the label were expected to click on the link. Thus, only ‘yes’ responses were expected although a few individuals did indicate ‘no’. The behavioral experiment emails were sent out 6 days before the online survey was released to lower the perception that the email experiment was directly related to the survey. Only responses to the behavioral experiment emails received before the survey was launched were included in the analysis. Responding to the email experiment was not a prerequisite for filling in the subsequent online survey.

### The online survey and regression analysis

The online survey asked respondents to identify the factors that motivate them to consider an energy label. The questionnaire was paired with actual energy consumption data in order to contrast individuals’ actual and desired energy behavior. The questionnaire was designed to collect information on: (1) self-identified motivational factors behind energy label adoption; (2) socio-demographic characteristics of respondents; and (3) consumers’ perceived monetary value or WTP for the label. Several questions aimed to capture the extent of egoistic, biospheric and social normative concerns across respondents as well as their trust in the energy label and local/government institutions as a source of information. Participants were probed on their perceived level of responsibility towards (1) resource depletion, (2) climate change and (3) general environmental problems, as well as their perceived level of energy efficiency. For several questions, we used a five (or seven) point Likert scale to measure the level of agreement with a given statement (see Albaum 1997). For example, respondents were questioned on the importance they attach to increasing their property value or lowering costs when deciding on energy efficiency on a 1–5 Likert scale (with larger values corresponding to higher importance). Appendix Table 6 provides a description of all variables, the scale at which they were measured, as well as references to the original studies that motivated us to include them in our analysis—for all variables we have adopted the same, or very similar, Likert scale as the ones used in these original studies. Respondents were also asked about their perceived WTP for an energy label. As customary in the literature, a payment card with 22 optional values (between 0 and more than €1000) accompanied the WTP question (based on Rowe et al. 1996, which assumes that people’s ability to perceive differences decreases as the value increases). However, individuals were not constrained by this range and could opt for any possible positive value.

To explain the variation in WTP across respondents and its association with several respondent (and housing) specific characteristics, we performed a multivariate regression analysis estimating 10 different specifications varying the set of explanatory variables (see Table 4). The first specification only included socio-demographic variables (column 1). While keeping the socio-demographic variables in the analysis, we consecutively included in our empirical specification: building and tenancy characteristics (column 2), past and future renovation plans (column 3), perceived social responsibility for environmental problems (column 4), perceived ability to improve energy efficiency (column 5), perceived negative side-effects of energy conservation (column 6), perceived positive side-effects of energy conservation (column 7), trust (column 8) and a combination of all factors used for the regression analysis (column 9). In column 10 we only included explanatory variables that were found to be statistically significant in at least one of the first eight specifications tested. Regression analysis is a common statistical technique used to estimate the relationship between a dependent variable (WTP in this case) and a set of explanatory factors. The estimated coefficient for each explanatory variable corresponds to the predicted change in WTP for each one-unit difference in this particular explanatory variable (assuming that all other explanatory variables of the model are held constant). There is a detailed description of all of the variables in Appendix Table 6 and their descriptive statistics in Table 7.

## Results

### Behavioral email experiment

A total of 317 energy consumers stated that ‘yes’ they were interest in receiving an energy label (from the sample of 3188 clients emailed; i.e. the four different message groups of 797 individuals each). The *neutral* frame received more positive responses (102) than any other category. The *social norms* frame had the second highest response rate (84), which was significantly higher compared to the *biospheric* framed message (and slightly higher than the *egoistic* framed message). A Pearson Chi square test [c^2^ (3, *N* = 3188) = 15.96 *p* = .00] signified that these differences across the four motivational frames are statistically significant. Summary results are presented in Table 3. This is in line with earlier research stressing that neutrally framed messages are often more persuasive compared to messages that convey some form of obligation (see Kolyesnikova et al. 2011). Although social norms did not score the highest, the relative high response attributed to the social norm messages is consistent with earlier research by Goldstein et al. (2008). We also examined statistical differences in the positive response (‘yes’ reply to get energy label) across pairs of motivational frames. The statistical significance of the difference in the response rate is the highest for the *neutral* information and *biospheric* groups, followed by the *biospheric* and *social norms*.

**Table 3:** Email experiment (responses)

Email groups and intent to get an energy label		Energy label		No. email recipients
		No	Yes	
*Group*				
Egoistic (self-interest)	Count	4	76	797
Biospheric (environment)	Count	7	55	797
Social norms	Count	5	84	797
Neutral information	Count	0	102	797
Total	Count	16	317	3188

	Value	Degrees of freedom	Asymptotic significance
Pearson Chi square	15.96	3	0.00

### The online survey and regression analysis

Our results (based on the primary data collected from the online survey) indicate that the average participant has a stronger than neutral feeling of responsibility for environmental problems [i.e. the average value for the level of responsibility for environmental problems is 3.66 in a 1 (no responsibility) to 5 (full responsibility) scale], see correlation table in Appendix Table 8. However, no significant correlation is detected between the perceived level of environmental responsibility and the actual gas consumption level. The results also illustrate that individuals have low expectations with respect to the energy label’s added value. Most individuals (53 %) stated that they would only get a label if it were mandatory, while few individuals (<35 %) cited the usefulness of getting a residential energy label to save energy.

**Fig. 2:**
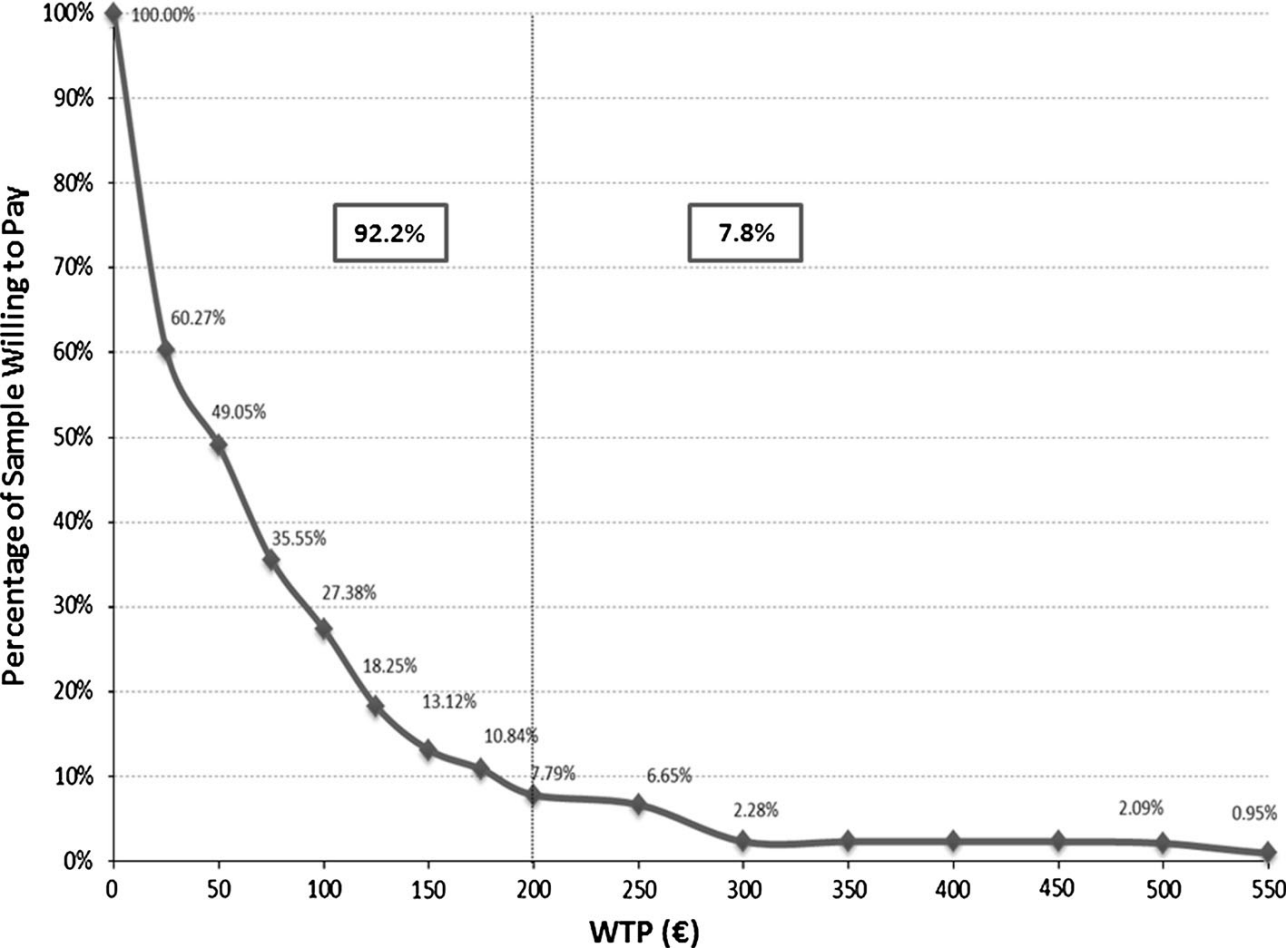
Percentage of respondents willing to pay a given price for the label

On average, the participants’ perceived value of an energy label is low. The average respondent’s WTP for an energy label (for the 526 consumers, out of the 611 surveyed, who answered this particular question and, hence, provided the estimates) is €74.11 (more than 70 % lower than the average market price before 2015, approximately €200). Figure 2 displays the cumulative distribution of the sample in terms of stated WTP. Only approximately 8 % of the respondents are willing to pay the current average market price for an energy label. Almost 40 % of the sample is not willing to pay more than €25, while approximately 29 % of the sample opted for a zero value (i.e. no WTP).

The regression results of Table 4 indicate that age was the only socio-demographic factor that was consistently a significant predictor of WTP (a quadratic specification of age was also tested but was found to be statistically insignificant). Thus, the older people are, the more willing they are to invest in an energy label. This is in line with earlier evidence from the literature suggesting that older individuals often tend to be more interested in energy conservation (Brounen et al. 2011). We included the attribute renters and homeowners in the WTP regressions, but the results showed little difference between the two groups. This result should be interpreted with caution due to the small sample size of renters who participated in the survey. For this reason, this attribute was not analyzed separately in the WTP regression analysis. In the Netherlands, many individuals rent long-term, often through social housing corporations. Given their long-term occupancy, it is perhaps not surprising that their behavior can resemble the behavior of actual owners (e.g. installing floors and maintenance).

**Table 4:** Regression analysis—WTP and respondent/housing characteristics

Independent variable	WTP (1)	WTP (2)	WTP (3)	WTP (4)	WTP (5)	WTP (6)	WTP (7)	WTP (8)	WTP (9)	WTP (10)
(Constant)	−40.04 (−1.19)	−4.42 (−0.10)	−68.63 (−1.26)	−109.33** (−2.38)	−130.800*** (−2.60)	−115.95** (−2.20)	−96.21 (−1.63)	−182.17*** (−3.03)	−236.21* (−1.83)	−153.49*** (−2.55)
Gender	2.33 (0.21)	5.28 (0.39)	4.14 (0.30)	7.06 (0.64)	2.74 (0.24)	5.16 (0.46)	6.49 (0.58)	10.85 (0.85)	13.66 (0.76)	
Age	14.72*** (3.53)	17.40*** (3.14)	16.19*** (3.08)	13.30*** (3.21)	15.78*** (3.62)	13.93*** (3.20)	12.89*** (2.89)	19.41*** (3.99)	9.77 (1.14)	19.17*** (3.64)
Size household	4.30 (1.02)	3.33 (0.66)	8.03 (1.45)	3.58 (0.39)	2.85 (0.67)	4.57 (1.08)	4.96 (1.15)	4.26 (0.90)	6.93 (1.01)	
Income	5.57 (1.42)	7.46 (1.50)	0.82 (.16)	5.22 (1.32)	6.74* (1.69)	5.27 (1.31)	4.66 (1.13)	7.56* (1.66)	1.59 (0.23)	7.63* (1.78)
Education	1.26 (0.41)	−3.51 (−0.91)	4.57 (1.16)	−0.26 (−0.08)	0.62 (0.19)	0.08 (0.03)	0.31 (0.10)	−1.73 (−0.64)	−2.77 (−0.50)	
Planned length of occupancy		−10.71*** (−3.08)						−10.35*** (−3.14)	−8.50* (−1.72)	−10.72*** (−2.88)
Type of house		−2.51 (−0.50)							−8.40 (−1.28)	
Period built		−1.89 (−0.55)							−4.16 (−0.91)	
Perceived gas use		6.83 (0.28)							14.18 (1.54)	
Actual gas use		0.01 (0.58)							0.01 (0.92)	
Family renovated			−2.11 (−0.15)						3.50 (0.21)	
Friends renovated			−26.80* (−1.89)						−20.68 (−1.20)	−21.13 (−1.55)
Neighbors renovated			25.38* (1.81)						34.35* (1.91)	14.44 (1.03)
Respondent plans to renovate			9.55 (0.76)						17.17 (0.99)	
Feel responsible for resource depletion				9.13 (1.09)					18.49 (1.26)	
Feel responsible for GHG				12.28* (1.73)					7.38 (0.59)	10.77* (1.66)
Feel responsible for environmental problems				−7.24 (−0.95)					−6.54 (−0.55)	
Believe energy used efficiently				−1.83 (−0.33)					−2.41 (−0.24)	
Can save 5 % of energy use					5.40 (0.88)				−8.69 (−0.87)	
Can save 20 % of energy use					9.07 (1.57)				17.02* (1.90)	
Want to save energy in the future					−3.35 (−0.35)				−7.40 (−0.78)	
No idea how to save energy					2.62 (0.59)				−3.30 (−0.44)	
Importance of energy savings				7.72 (1.08)	11.84* (1.67)	18.50*** (2.64)	14.54* (1.91)	14.68* (1.84)	16.97 (1.30)	12.06 (1.34)
Conservation: is a lot of work						7.48 (1.34)			5.13 (0.58)	
Conservation: less comfortable life						−1.47 (−0.24)			6.75 (0.65)	
Conservation: loss of freedom						3.62 (0.53)			9.19 (0.78)	
Conservation: is time consuming						−4.71 (−0.84)			−6.63 (−0.73)	
Conservation: unnecessary						−2.59 (−0.58)			5.95 (0.73)	
Reason: improve environment							5.08 (0.58)		0.71 (0.04)	
Reason: make house more comfortable							0.59 (0.09)		−8.17 (−0.81)	
Reason: increase house value							2.88 (0.50)		3.76 (0.40)	
Reason: lower costs							−9.91 (−1.13)		2.46 (0.17)	
Reason: lessen CO_2_ emissions							−0.71 (−0.09)		−12.40 (−0.99)	
Reason: less sensitive to price increases (energy)							3.26 (0.42)		−4.33 (−0.36)	
Trust environmental groups								11.86** (2.01)	7.74 (0.85)	7.24* (1.03)
Trust energy label								14.23** (2.12)	18.76* (1.94)	14.51** (1.93)
Trust local and national government								2.68 (0.38)	4.49 (0.40)	
R^2^	0.05	0.10	0.07	0.09	0.08	0.07	0.07	0.15	0.28	0.17
N	369	266	260	367	354	363	365	292	184	233

***, **, * correspond to 1, 5 and 10 % level of significance. T-statistics are provided in parenthesis. Five outliers were excluded, where responses on WTP were €700 and above)

As one would expect, those who are occupying more recently built properties have a lower WTP, but the correlation is not statistically significant. Surprisingly, how long individuals plan to stay in the same home (planned occupancy duration) is negatively correlated with WTP (despite the fact that longer occupancy can provide an opportunity for higher accumulated benefits as a result of energy conservation; see Table 4 columns 2, 8, 9 and 10). However, this negative correlation can be attributed to the policy in place in the Netherlands of only requiring a residential energy label when buying, selling or renting a house. Many renters are unaware whether their residence has a label and what the label score is. Neither the perceived nor actual gas consumption is statistically significant in explaining the variation in WTP (although higher energy consumption is associated with a higher WTP). Having neighbors who financed an important renovation of their house in the previous year (costing more than €1000) appears to be a motivating factor behind adopting an energy label (columns 3 and 9). There is ample evidence in the literature pointing to the role of social connectivity (and particularly learning from neighbors) in diffusion of certain practices and technologies (e.g. Goldstein et al. 2008; Manski 2000).

As expected, the degree of importance attached to energy savings correlates positively [although weakly for specifications (4), (8) and (9)] with the WTP level for an energy label (see Table 4). In terms of assuming responsibility for the environment, only a sense of responsibility for minimizing GHGs significantly explains WTP for energy labels (columns 4 and 10). The perceived ability to contribute to energy conservation as well as the motivating factors related to either negative or positive side-effects of energy label adoption appear to have weak statistical power in explaining WTP levels. In other words, respondents do not strongly associate a residential energy label with specific energy conservation goals they might wish to pursue (see columns 5–7). On the other hand, trust in environmental organizations and the energy label as sources of information for energy conservation is positively associated with increased WTP (by €11.86 and €14.23 respectively; see column 8). This is in line with empirical work pointing to a close relationship between self-efficacy (own ability to reach goals) and trust in institutions (e.g. there is much evidence suggesting that trust in information renders individuals more proactive and raises the rate of adoption of certain technologies—e.g. see Lee and Lin (2009) and Bélanger and Carter (2008) for the role of trust in influencing behavior and technology adoption in medicine and the IT sector. Blake (1999) also claims that lack of trust is often one of the key barriers behind limited pro-environmental behavior.

We also tested whether the WTP for an energy label varies significantly across the five different groups: i.e. the four groups who received the differently framed messages of the email experiment, as well as the fifth group that only participated at the online survey (see Table 5). There was a significant effect at the *p* < .10 level of the different message framing on WTP [*F*(4, 521) = 2.20, *p* = .068]. The group that received the *biospheric* message expressed the highest WTP (€105.38), while the group that received the *egoistic* message expressed the lowest average WTP (€60.11). Hence, while messages with a *biospheric* motivation triggered the lower response rates in our email experiment, those who received them expressed the highest average WTP levels for residential energy labels. Both the groups that received the *egoistic* and *neutral* message types have lower average WTP than the group that did not receive the email experiment.

**Table 5:** Comparison of average WTP across five groups

Group	Mean	N	Std. deviation
Egoistic	60.11	108	108.38
Biospheric	105.38	93	166.50
Social norms	74.34	94	123.06
Neutral information	65.96	116	85.44
Group not participating in the email experiment	69.99	115	100.45
Total	74.11	526	118.14

ANOVA [*F*(4, 521) = 2.20, *p* = .068]

## Discussion

The study sets out to assess the role of different factors in motivating energy conservation through energy labels. First, we demonstrate that *how* a message is framed can affect the response rate. In our email experiment, *neutral* information performed the best in promoting interest in the residential energy label. This suggests that the *neutral* framing of the message may improve the credibility of energy labels, particularly considering their negative past publicity. Nonetheless, *social norms* can still play a role in motivating people to get an energy label given their relatively high response rate and WTP. At the same time, although the goal is to promote environmental outcomes, this study indicates that Dutch households are the least motivated, in terms of response rate, to get a label when the *biospheric* frame is applied. The *egotistic* frame, traditionally used in Dutch governmental information campaigns (De Groot and Steg 2009), had a relatively low response rate along with the lowest WTP. The findings suggest that policymakers need to consider alternative approaches to the traditional *egotistic* framing or exclusive dependence on *biospheric* framing in order to increase response levels.

The study’s findings suggest that, in general, the response rate to a message framing is not indicative of the WTP level (i.e. a higher response rate does not mean a higher WTP). The results indicate that those who received *neutral* framed or *social norms* based messages (compared to *biospheric* ones) had the highest response rates but not the highest WTP. The group that received the *biospheric* message expressed the highest WTP (€105.38), while the group that received the *egoistic* message expressed the lowest average WTP (€60.11), but had a relatively low response rate. Different groups have different average WTP, but of course label providers offer clients a uniform price—this suggests that the success of the energy label initiative would require appropriately framed messages in combination with a pricing scheme (of sufficiently low rates) that takes into consideration the differences in WTP across groups/messages.

A socio-demographic factor contributing to high WTP is age. These results are in line with those obtained by Brounen et al.’s (2011) that young (and high-income) individuals have the lowest awareness levels of energy consumption. Making energy conservation more meaningful for young people is an important issue for future research. Policymakers should realize that younger energy consumers have both lower awareness and are more resistant to paying for an energy label.

We observe a high level of resistance to paying for an energy label, as indicated by the 28.7 % of respondents who opted for a zero WTP for a label. As of 2015, the provisional label is free and the definite label costs ‘a few bucks’ (Rijksoverheid 2015). The changes made in 2015 are better aligned with peoples’ WTP shown in this study. However, although the current label has the benefits of low cost, the accuracy and added value of the information provided are questionable as indicated by previous research (Backhaus et al. 2011; Brounen and Kok 2011a; Majcen et al. 2013a, b). The cheaper price comes at the expense of not having a personalized inspection of the property’s energy efficiency nor tailored recommendations.

A possible explanation for the resistance to pay for the label is that few individuals find the label useful. Contrary to expectations, the findings suggest that the length of occupancy is negatively correlated to WTP for an energy label. Many respondents express an interest in having a label only when mandatory, while few respondents acknowledge any perceived potential benefits of the label. The current policy focuses on those selling or renting to others. However, this is not the group that is likely to make improvements to the energy efficiency of buildings while renovating. Greater efforts are needed to ensure that long-term occupants, who have the potential to benefit from improved energy efficiency, use the label. Clear communication of the type of information the energy label provides and the anticipated benefits of residential energy labels (that accrue to individuals, the natural environment as well as the society as a whole) could incentivize long-term occupants to use the label.

However, the amended energy label policies still focus on mandatory requirements (which can be opted out of up until the last moment) instead of improving the label’s usefulness and matching consumers’ interests with energy conservation. The energy label program’s goal is to lower emissions through energy conservation. However, unless individuals are motivated to use the label’s information to improve energy efficiency in the housing stock, the label remains a window dressing instead of aiding energy conservation.

## Appendix

See Tables 6, 7 and 8.

**Table 6:** Variables description

WTP	Question asked how willing the individual is to pay for an energy label if it is mandatory and has the purpose of informing the individual of their house’s energy efficiency and of giving advice on how to improve the energy efficiency. Values on payment card from €0 to €1000
Gender	Discrete: female, male
Age	6-point scale: <20; 21–30; 31–40; 41–50; 50–65; 65+
Size household	Number of people in household including respondent Interval scale: 1, 2, 3, 4, 5+
Income	Continuous scale of gross income with option not to answer €0–1000; €1000–2000; €2000–3000; €3000–4000; €4000–5000; €5000+
Education level	Based on the Dutch educations system 12-point scale from lower to higher educational attainment
Planned length of occupancy	The number of years the individual plans to remain at the current address Scale: <2 years; 2–5 years; 6–10 years; 11–15 years; 16–20 years; more than 20 years; I don’t know
Type of house	The type of house the respondent lives in, going from a structure that has walls with the least amount of exposure to outer climate to the most Apartment; row house; corner house; duplex; free standing house
Period built	Period of house built. Buildings constructed during the same period generally had the same level of insulation Discontinuous scale: before 1919; 1919–1945; 1946–1970; 1971–1980; 1981–1990; 1991–2000; after 2000; I don’t know
Perceived gas use	Question asked whether the respondent thinks that they consume more or less gas than the Dutch average of 1600 m^3^ per year Likert scale (1–5): much less than average; less than average; average; more than average; much more than average; I don’t know
Actual gas use	Value is respondent’s gas consumption for the past year (m^3^/a)
Actual electricity use	Value is respondent’s electricity consumption for the past year (kWh/a)
Actual label score	Only respondents who answered ‘yes’ to whether they had an energy label were asked this question. The respondent was asked what rating their house received from the existing label scale of A–G where A denotes an energy efficient house and G denotes a very inefficient house. Scale: (1–7, where A = 1, B = 2)
Perceived label score	Respondents who answered ‘no’ or ‘maybe’ to whether they had an energy label were asked this question. Respondent were asked what rating their house received from the existing label scale of A–G where A denotes an energy efficient house and G denotes a very inefficient house. Scale: (1–7, where A = 1, B = 2)
Family renovated	Question asked whether a family member financed a renovation for more than €1000 in the past 12 months. Inspired by Adjei et al. (2012) Yes; no; I don’t know
Friends renovated	Question asked whether a friend financed a renovation for more than €1000 in the past 12 months. Inspired by Adjei et al. (2012) Yes; no; I don’t know
Neighbors renovated	Question asked whether a friend financed a renovation for more than €1000 in the past 12 months. Inspired by Adjei et al. (2012) Yes; no; I don’t know
Respondent plans to renovate	Question asked whether the respondent expects to renovate in the next 3 years. Inspired by Adjei et al. (2012) Yes; no
Feel responsible for resource depletion	Question asked to what degree the respondent dis/agrees with the statement that their energy consumption contributes to resource depletion. Based on research by Abrahamse and Steg (2009; 2011) on the level of perceived responsibility for environmental problems stating that attitude and values are important indicators of environmental conservation Likert scale 1–5: completely disagree to completely agree
Feel responsible for GHG	Question asked to what degree the respondent dis/agrees with the statement that their energy consumption contributes to GHG. Based on research by Abrahamse and Steg (2009, 2011) on the level of perceived responsibility for environmental problems stating that attitude and values are important indicators of environmental conservation Likert scale 1–5: completely disagree to completely agree
Feel responsible for environmental problems	Question asked to what degree the respondent dis/agrees with the statement that their energy consumption contributes to general environmental problems. Based on research by Abrahamse and Steg (2009, 2011) on the level of perceived responsibility for environmental problems stating that attitude and values are important indicators of environmental conservation Likert scale 1–5: completely disagree to completely agree
Believe energy used efficiently	Question asked to what degree the respondent dis/agrees with the statement that they already efficiently consume energy. Based on research by Abrahamse and Steg (2009, 2011) on the level of perceived responsibility for environmental problems stating that attitude and values are important indicators of environmental conservation and by Cialdini and Schultz (2004) Likert scale 1–5: completely disagree to completely agree
Can save 5 % of energy use	Question asked to what degree the respondent dis/agrees with the statement that it would be easy to save 5 % of their household consumption. Refers to the concept of perceived behavioral control inspired by research by Abrahamse and Steg (2011) Likert scale 1–5: completely disagree to completely agree
Can save 20 % of energy use	Question asked to what degree the respondent dis/agrees to the statement that it would be easy to save 20 % of their household consumption. Refers to the concept of perceived behavioral control, specifically how capable they feel they are for conserving energy, inspired by research by Abrahamse and Steg (2011) Likert scale 1–5: completely disagree to completely agree
Want to save energy in the future	Question asked to what degree the respondent dis/agrees to the statement that they want to lower their future energy consumption. Refers to the concept of perceived behavioral control inspired by research by Abrahamse and Steg (2011) Likert scale 1–5: completely disagree to completely agree
No idea how to save energy	Question asked to what degree the respondent dis/agrees to the statement that they do not know how they could use less energy. Refers to the concept of perceived behavioral control inspired by research by Abrahamse and Steg (2011) Likert scale 1–5: completely disagree to completely agree
Importance of energy savings	Inspired by value-belief-norms theory on how values motivate energy conservation behavior Steg and Vlek (2009), Stern (2000) and work by Cialdini and Schultz (2004) Likert scale 1–5: entirely unimportant to completely important
Conservation: is a lot of work	Question asked to what degree the respondent dis/agrees to the statement that energy conservation is a lot of work in order to gauge the respondent’s perception of energy saving Abrahamse and Steg (2009, 2011) Likert scale 1–5: completely disagree to completely agree
Conservation: less comfortable life	Question asked to what degree the respondent dis/agrees to the statement that energy conservation means experiencing less living comfort in order to gauge the respondent’s perception of energy saving Abrahamse and Steg (2009, 2011) Likert scale 1–5: completely disagree to completely agree
Conservation: loss of freedom	Question asked to what degree the respondent dis/agrees to the statement that energy conservation limits personal freedom in order to gauge the respondent’s perception of energy saving Abrahamse and Steg (2009, 2011) Likert scale 1–5: completely disagree to completely agree
Conservation: is time consuming	Question asked to what degree the respondent dis/agrees to the statement that energy conservation costs too much time to realize energy savings in order to gauge the respondent’s perception of energy saving Abrahamse and Steg (2009, 2011), Cialdini and Schultz (2004) Likert scale 1–5: completely disagree to completely agree
Conservation: unnecessary	Question asked to what degree the respondent dis/agrees to the statement that they have already done everything to conserve energy in order to gauge the respondent’s perception of energy saving Abrahamse and Steg (2009, 2011) Likert scale 1–5: completely disagree to completely agree
Reason: improve environment	Question asked how important is improving the environment to the individual as a benefit of making their home more energy efficient. Inspired by personal norm and motivation research by Cialdini and Schultz (2004) and Sütterlin et al. (2011) Likert scale 1–5: completely unimportant; unimportant; neutral; important; very important
Reason: make house more comfortable	Question asked how important is making their home more comfortable to the individual as a benefit of making their home more energy efficient. Inspired by personal norm and motivation research by Cialdini and Schultz (2004) and Sütterlin et al. (2011) Likert scale 1–5: completely unimportant; unimportant; neutral; important; very important
Reason: increase house value	Question asked how important is improving the house’s value to the individual as a benefit of making their home more energy efficient. Inspired by personal norm and motivation research by Cialdini and Schultz (2004) and Sütterlin et al. (2011) Likert scale 1–5: completely unimportant; unimportant; neutral; important; very important
Reason: lower costs	Question asked how important is lowering energy costs to the individual as a benefit of making their home more energy efficient. Inspired by personal norm and motivation research by Cialdini and Schultz (2004) and Sütterlin et al. (2011) Likert scale 1–5: completely unimportant; unimportant; neutral; important; very important
Reason: lessen CO_2_ emissions	Question asked how important is lowering CO_2_ emissions to the individual as a benefit of making their home more energy efficient. Inspired by personal norm and motivation research by Cialdini and Schultz (2004) and Sütterlin et al. (2011) Likert scale 1–5: completely unimportant; unimportant; neutral; important; very important
Reason: less sensitive to price increases (energy)	Question asked how important is being less sensitive to higher energy prices in the future to the individual as a benefit of making their home more energy efficient. Inspired by personal norm and motivation research by Cialdini and Schultz (2004) and Sütterlin et al. (2011) Likert scale 1–5: completely unimportant; unimportant; neutral; important; very important
Trust environmental groups	Question asked how trustworthy the respondent found environmental groups as a source of information for personal energy consumption. Based on the survey conducted by ECN Adjei et al. (2012) Likert scale 1–5: completely untrustworthy to completely trustworthy
Trust energy label	Question asked how trustworthy the respondent found the energy label as a source of information for personal energy consumption. Based on the survey conducted by ECN (Adjei et al. 2012) Likert scale 1–5: completely untrustworthy to completely trustworthy
Trust local and national government	Question asked how trustworthy the respondent found the local and national government as a source of information for personal energy consumption. Based on the survey conducted by ECN Adjei et al. (2012) Likert scale 1–5: Completely untrustworthy to completely trustworthy

**Table 7:** Descriptive statistics

Variable	Number	Mean	Minimum	Maximum	Standard deviation
WTP	526	74.11	0	1000	118.14
Gender	534	1.66	1	2	.473
Age	536	4.40	2	6	1.12
Size household	537	2.66	1	5	1.17
Income	391	4.32	1	6	1.46
Education	523	6.35	1	8	1.75
Planned length of occupancy	486	3.81	1	6	1.67
Type of house	608	3.20	1	5	1.31
Period built	601	3.76	1	7	1.75
Perceived gas use	548	3.19	1	5	1.14
Actual gas use	4000	1904.60	746.49	1001	8142
Actual electricity use	4000	3523.24	1416.46	512	6996
Actual label score	51	3.55	1	7	1.85
Perceived label score	525	3.38	1	7	1.47
Family renovated	492	1.59	1	2	0.49
Friends renovated	446	1.61	1	2	0.49
Neighbors renovated	473	1.65	1	2	0.48
Respondent plans to renovate	589	1.69	1	2	0.46
Feel responsible for resource depletion	580	3.85	1	5	1.10
Feel responsible for GHG	580	3.59	1	5	1.16
Feel responsible for environmental problems	578	3.66	1	5	1.15
Believe energy used efficiently	580	3.80	1	5	0.88
Can save 5 % of energy use	560	3.83	1	5	1.07
Can save 20 % of energy use	559	2.44	1	5	1.08
Want to save energy in the future	559	3.90	1	5	0.99
No idea how to save energy	558	2.41	1	5	1.16
Degree value energy savings	572	4.38	1	5	0.73
Conservation: is a lot of work	566	2.10	1	5	0.97
Conservation: less comfortable life	567	2.06	1	5	0.97
Conservation: loss of freedom	568	1.95	1	5	0.89
Conservation: is time consuming	566	2.32	1	5	0.95
Conservation: unnecessary	569	2.96	1	5	1.04
Reason: improve environment	560	4.04	1	5	0.73
Reason: make house more comfortable	559	3.87	1	5	0.74
Reason: increase house value	560	3.49	1	5	0.88
Reason: lower costs	559	4.18	1	5	0.65
Reason: lessen CO_2_ emissions	559	3.93	1	5	0.81
Reason: less sensitive to price increases (energy)	558	4.06	1	5	0.72
Trust environmental groups	544	3.30	1	5	0.99
Trust energy label	545	3.43	1	5	0.86
Trust local and national government	540	3.40	1	5	0.86

**Table 8:** Correlation table

	Spearman’s rho		(1)	(2)	(3)	(4)	(5)	(6)	(7)	(8)	(9)
(1)	Feel responsible for resource depletion	Coefficient	1.00								
		Sig. (2-tailed)	.								
		N	58								
(2)	Feel responsible for GHG	Coefficient	0.81**	1.00							
		Sig. (2-tailed)	0.00	.							
		N	580	580							
(3)	Feel responsible for environmental problems	Coefficient	0.81**	0.85**	1.00						
		Sig. (2-tailed)	0.00	0.00	.						
		N	578	578	578						
(4)	Believe energy used efficiently	Coefficient	0.29**	0.28**	0.27**	1.00					
		Sig. (2-tailed)	0.00	0.00	0.00	.					
		N	579	579	577	580					
(5)	Actual label score	Coefficient	−0.07	−0.01	0.03	−0.15	1.00				
		Sig. (2-tailed)	0.61	0.93	0.84	0.31	.				
		N	51	51	51	50	51				
(6)	Perceived label score	Coefficient	0.01	0.01	0.06	−0.14**	.	1.00			
		Sig. (2-tailed)	0.93	0.87	0.18	0.01	.	.			
		N	522	522	520	522	0	525			
(7)	Actual gas use	Coefficient	0.02	0.02	0.01	−0.12**	0.31*	0.16**	1.00		
		Sig. (2-tailed)	0.67	0.63	0.93	0.01	0.02	0.01	.		
		N	580	580	578	580	51	525	4001		
(8)	Actual electricity use	Coefficient	−0.09	−0.07	−0.09	−0.30**	−0.11	−0.09*	0.42**	1.00	
		Sig. (2-tailed)	0.04	0.08	0.07	0.01	0.44	0.04	0.01	.	
		N	580	580	578	580	51	525	4001	4001	
(9)	Perceived gas use	Coefficient	−0.02	−0.01	−0.02	−0.23**	0.27	0.27**	0.76**	0.41**	1.00
		Sig. (2-tailed)	0.64	0.77	0.66	0.00	0.06	0.00	0.00	0.00	.
		N	522	522	520	522	48	472	548	548	548
(10)	Cans save 5 % of energy use	Coefficient	0.23**	0.20**	0.22**	0.07	−0.10	0.12**	−0.01	−0.02	0.04
		Sig. (2-tailed)	0.00	0.00	0.00	0.08	0.48	0.01	0.90	0.63	0.39
		N	560	560	558	559	48	508	560	560	505
(11)	Can save 20 % of energy use	Coefficient	0.17**	0.16**	0.18**	0.02	−0.11	0.14**	−0.01	0.01	−0.01
		Sig. (2-tailed)	0.00	0.00	0.00	0.68	0.45	0.00	0.85	0.98	0.95
		N	559	559	557	558	48	507	559	559	504
(12)	Want to save energy in the future	Coefficient	0.22**	0.27**	0.26**	0.15**	−0.10	0.06	0.05	−0.03	0.06
		Sig. (2-tailed)	0.00	0.00	0.00	0.00	0.49	0.19	0.26	0.45	0.19
		N	559	559	557	558	49	506	559	559	504
(13)	Importance of	Coefficient	0.39**	0.35**	0.34**	0.29**	0.06	−0.01	0.04	−0.13**	−0.02
		Sig. (2-tailed)	0.00	0.00	0.00	0.00	0.66	0.97	0.37	0.00	0.63
		N	571	571	569	571	51	513	572	572	515
(14)	No idea how to save energy	Coefficient	−0.02	−0.03	0.01	−0.06	0.01	0.07	0.05	−0.03	0.08
		Sig. (2-tailed)	0.59	0.52	0.75	0.19	0.93	0.11	0.24	0.44	0.09
		N	557	557	555	557	48	505	558	558	503
(15)	Reason: improve environment	Coefficient	0.50**	0.48	0.52	0.21	0.02	0.04	−0.05	−0.166**	−0.09
		Sig. (2-tailed)	0.00	0.00	0.00	0.00	0.89	0.42	0.25	0.00	0.05
		N	560	560	558	559	51	503	560	560	505
(16)	Reason: lessen CO_2_ emissions	Coefficient	0.48**	0.49**	0.47**	0.23**	−0.02	−0.01	−0.02	−0.11**	−0.05
		Sig. (2-tailed)	0.00	0.00	0.00	0.00	0.87	0.79	0.73	0.019	0.23
		N	559	559	557	558	51	502	559	559	504
(17)	Age	Coefficient	−0.02	0.02	−0.02	0.04*	−0.32**	−0.03	0.28**	0.11*	0.15**
		Sig. (2-tailed)	0.62	0.64	0.63	0.41	0.03	0.46	0.00	0.01	0.00
		N	535	535	533	536	49	481	536	536	482

	Spearman’s rho		(10)	(11)	(12)	(13)	(14)	(15)	(16)	(17)
(10)	Cans save 5 % of energy use	Coefficient	1.00							
		Sig. (2-tailed)	.							
		N	560							
(11)	Can save 20 % of energy use	Coefficient	0.69**	1.00						
		Sig. (2-tailed)	0.00	.						
		N	556	559						
(12)	Want to save energy in the future	Coefficient	0.34**	0.33**	1.00					
		Sig. (2-tailed)	0.00	0.00	.					
		N	556	556	559					
(13)	Importance of	Coefficient	0.18**	0.14**	0.39**	1.00				
		Sig. (2-tailed)	0.00	0.00	0.00	.				
		N	555	554	554	572				
(14)	No idea how to save energy	Coefficient	−0.21**	−0.189**	0.01	0.02	1.00			
		Sig. (2-tailed)	0.00	0.00	0.90	0.68	.			
		N	554	554	553	553	558			
(15)	Reason: improve environment	Coefficient	0.16**	0.13**	0.28**	0 .39**	−0.01	1.00		
		Sig. (2-tailed)	0.00	0.00	0.00	0.00	0.81	.		
		N	547	546	546	560	544	560		
(16)	Reason: lessen CO_2_ emissions	Coefficient	0.15**	0.155**	0.27**	0.42**	0.05	0.69**	1.00	
		Sig. (2-tailed)	0.00	0001	0.00	0.00	0.26	0.00	.	
		N	546	545	545	559	543	559	559	
(17)	Age	Coefficient	−0.20**	−0.13**	−0.16**	0.03	0.11*	−0.04	0.06	1.00
		Sig. (2-tailed)	0.00	0.00	0.00	0.47	0.01	0.39	0.17	.
		N	521	520	520	536	521	534	533	536

** and * correspond to 1 and 5 % level of significance respectively
